# Atherosclerotic Cardiovascular Disease Risk Prediction Models in China, Japan, and Korea: Implications for East Asians?

**DOI:** 10.1016/j.jacasi.2025.01.006

**Published:** 2025-03-04

**Authors:** Patricia K. Nguyen, Dong Zhao, Tomonori Okamura, Hyeon Chang Kim, Nathan D. Wong, Eugene Yang

**Affiliations:** a Department of Medicine, Division of Cardiovascular Medicine, Stanford University, Stanford, California, USA; b Department of Epidemiology, Beijing Anzhen Hospital, Capital Medical University, Beijing Institute of Heart, Lung, and Blood Vessel Diseases, National Clinical Research Center for Cardiovascular Diseases, Beijing, China; c Department of Preventive Medicine and Public Health, Keio University School of Medicine, Tokyo, Japan; d Department of Preventive Medicine, Integrative Research Center for Cerebrovascular and Cardiovascular Diseases, Yonsei University College of Medicine, Seoul, Korea; e Heart Disease Prevention Program, Division of Cardiology, University of California, Irvine, Irvine, California, USA; f Division of Cardiology, Department of Medicine, University of Washington School of Medicine, Seattle, Washington, USA

**Keywords:** Asian immigrants, atherosclerosis, China, coronary artery disease, East Asia, Japan, Korea, stroke

## Abstract

The management of atherosclerotic cardiovascular disease (ASCVD) in the United States is currently based upon large epidemiological studies in primarily non-Hispanic White subjects. Although this strategy provides a uniform approach that is simpler to implement, it may result in inappropriately targeting certain Asian populations for treatment based on inaccurate ASCVD risk estimation. In this state-of-the-art review, we detail the similarities and differences in the prevalence of ASCVD and its risk factors among Chinese, Japanese, and Korean people living in the United States and in their native countries. We highlight the limitations of current risk calculators when applied to East Asian immigrants and summarize risk stratification approaches in China, Japan, and Korea. Our review underscores the need to disaggregate registry, cohort, and clinical trial data by East Asian subgroups, to actively engage these populations in research, and to initiate studies to better define ASCVD risk in East Asian people living in the United States.

## Highlights

• Prior studies have shown that the prevalence of ASCVD and its risk factors vary across East Asian countries, but whether these differences extend to East Asians living in the United States and how they affect the validity of ASCVD-risk calculators in East Asians in the United States and in their native countries remain unclear.
• ASCVD risk calculators, developed by the ACC/AHA, overestimate risk in Chinese, Koreans, and Japanese people. Risk calculators developed by professional organizations in the countries of origin, however, lack validation in East Asians living in the United States, who are exposed to different environmental and cultural influences.
• Future studies should include individuals from East Asian subpopulations and report disaggregated findings to inform the development of an ASCVD-risk calculator specific to East Asian U.S. immigrants that can then be prospectively validated.

East Asian people make up 20.7% of the world’s population based on the latest estimates by the United Nations.^1^ As the fastest growing immigrant population in the United States, Asians comprise approximately 7% of the U.S. population, with East Asians making up the largest Asian subgroup (eg, ∼40%). In 2019, cardiovascular disease (CVD) claimed 5.2 million lives in East Asian countries.^2^ Atherosclerotic cardiovascular disease (ASCVD), including ischemic heart disease, ischemic stroke, and peripheral arterial disease, was the leading cause of cardiovascular morbidity and mortality among East Asian persons.^2^ Declining fertility rates and increasing life expectancies will ensure that ASCVD continues to be one of the most common noncommunicable, chronic diseases affecting East Asian persons for future decades.^3^

Notably, epidemiological characteristics of ASCVD vary among East Asian persons compared with Southern, Western, and Central Asian persons.^2^ These differences exist even within East Asian subpopulations. The proportional mortality rate of CVD is as low as 25% in the Japanese and South Korean populations but as high as 40% in Chinese people, highlighting the need for both targeted and personalized, therapeutic strategies for East Asian subgroups.^2^

Whether the observed differences in the epidemiological characteristics in East Asian subgroups living in Asia extend to East Asian Americans is unknown. Because the number of East Asian immigrants living in the United States is growing rapidly, this question demands further investigation. In 2019 alone, 4.2 million East Asians immigrated to the United States, a sharp rise from the 250,000 who immigrated in 1960.^4^ Similar to those living in Asia, ASCVD is a major cause of morbidity and mortality. ASCVD risk varies widely between Asian American subgroups,^5^, ^6^, ^7^ underscoring the need to disaggregate data to refine risk assessment and optimize CVD management in East Asian Americans.

Cardiovascular clinical trials inclusive of East Asian Americans are limited. Like other Asian subgroups,^8^ East Asian U.S. immigrants and their offspring are not adequately represented among major U.S. prospective CVD cohort studies, which form the basis of the American College of Cardiology (ACC)/American Heart Association (AHA) ASCVD Pooled Cohort Equation (PCE) as well as other risk calculators. Although the recently developed AHA PREVENT (American Heart Association Predicting Risk of cardiovascular disease EVENTs) risk calculator, derived from a meta-analysis of 25 data sets, has removed race/ethnicity altogether arguing their effects may be already reflected in socioeconomic data,^9^ the accuracy of this calculator has not been specifically validated in East Asian persons. As a result, current risk stratification models are not adequate to predict the development of ASCVD in East Asian Americans. Whether ASCVD risk assessment tools based on long-term cohort studies conducted in East Asian countries can accurately predict risk in East Asian Americans is unknown. Although recalibration can create a useful ASCVD prediction model and has been proposed to improve accuracy, it is preferable to develop and validate a model using local data if available. Even among countries classified with similar risk levels, there are considerable differences in incidence rates of CVD. For example, in Korea, SCORE2-AP recalibrated SCORE2 to the Asia-Pacific region and categorized risk into 4 groups (low, moderate, high, and very-high risk). Korea is included in the low-risk group, but this low-risk regional model also overestimates risk in Korea.^10^ Additionally, the distribution of CVD subtypes varies by region and period, so the coefficients may differ as well. For example, although the coefficients for each predictor are somewhat similar, regional differences between models still exist. Thus, risk calculators that include the most recent local data would be the most appropriate. These knowledge gaps highlight the need for a more thorough understanding of variations in ASCVD disease burden and cardiovascular risk factor prevalence among East Asian people residing in the United States to improve risk stratification and management strategies.

In this review, we highlight the similarities and differences in the epidemiology, diagnosis, and treatment of ASCVD for individuals of East Asian origin who immigrated to the United States and their offspring (“East Asian Americans”) compared with those living in East Asia (“East Asian natives”). We identify major knowledge gaps in our understanding of ASCVD risk and explore opportunities and strategies to close these gaps through future clinical and research initiatives. Because of the lack of data and smaller size of other East Asian countries, this review focuses on studies performed in East Asian persons living or originating from China, Japan, and the Republic of Korea (South Korea).

## ASCVD Prevalence and Risk Factors in East Asian Populations

### Definition of East Asian populations

Based on definitions provided by the United Nations, the geographic boundaries of the Asia-Pacific region include Turkey in the west, the Pacific Island of Kiribati in the east, the Russian Federation in the north, to New Zealand in the south.^11^ Located in the Asia-Pacific region, major East Asian countries include the People’s Republic of China (China, including the special administrative regions of Hong Kong, Macao, and Taiwan), Democratic People’s Republic of Korea (North Korea), Republic of Korea (South Korea), Mongolia, and Japan. The major ethnic groups in East Asia include Han, Korean, and Yamato. Minor groups include Bai, Hui, Tibetan, Turkic, Manchus, Ryukyuan, Ainu, Zhuang, Mongols, and many other groups. In 2023, approximately 1.66 billion people live in East Asia, comprising 22% of the world’s population.^1^

Additionally, millions of East Asian immigrants and their offspring reside outside of the geographical boundaries of East Asia. Since the mid-1960’s, the number of immigrants from East Asia to the United States has risen sharply. According to calculations by the Migration Policy Institute of U.S. Census Data,^4^ between 2000 and 2019, the number of East Asian persons living in the United States has increased by 81% in the last 2 decades, outpacing Hispanic and non-Hispanic Black persons, whose numbers grew by 70% and 20%, respectively, during the same time frame. Immigrants from China and Korea accounted for 19% and 7% of the total Asian immigrant population, respectively.^4^

### Epidemiology of ASCVD in East Asian populations living in Asia and in the United States

An aging population together with the exponential rise in obesity and diabetes will continue to fuel the higher incidence of ASCVD among East Asian Americans and East Asian natives. Below, we summarize and compare the existing data on the epidemiology of ASCVD in East Asian natives and East Asian Americans.

**Figure 1:**
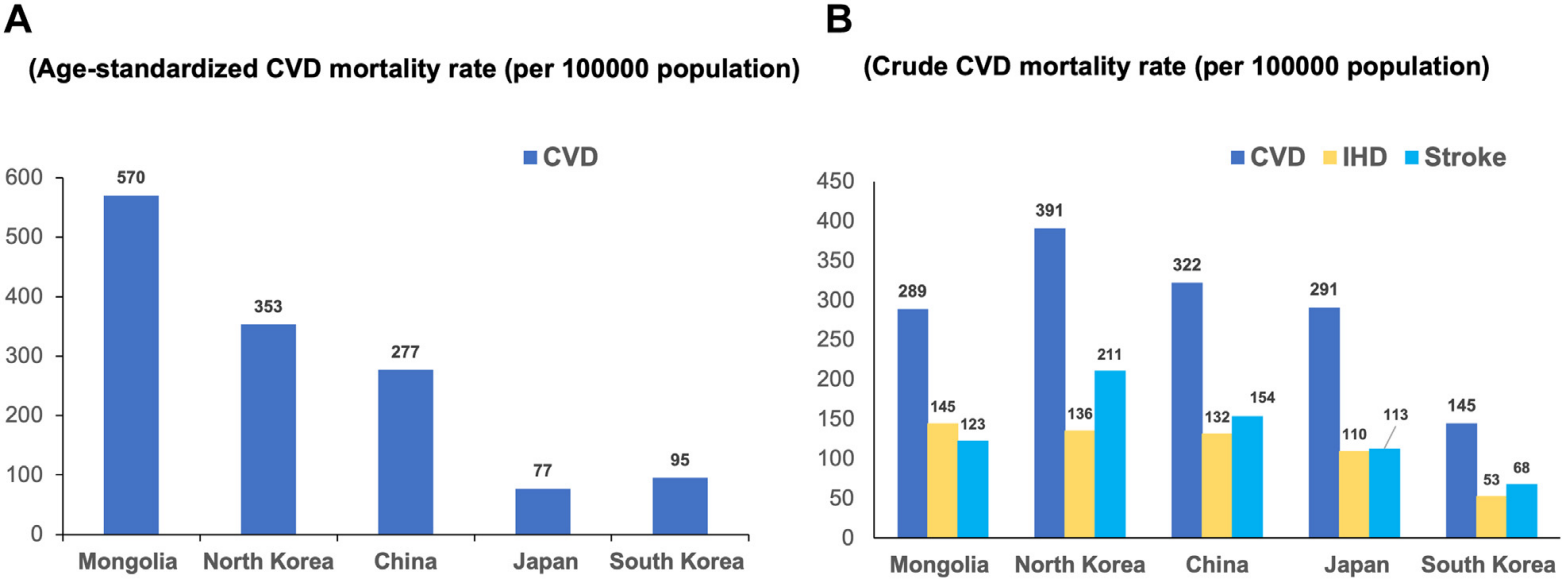
Age-Standardized and Crude Mortality Rates for Major CVD (2019) (A) Age-standardized cardiovascular disease (CVD) mortality rate (per 100,000 population). (B) Crude CVD mortality rate (per 100,000 population). Data was obtained from the open database of the Global Burden of Disease Study in the Global Health Data Exchange. IHD = ischemic heart disease.

**Figure 2:**
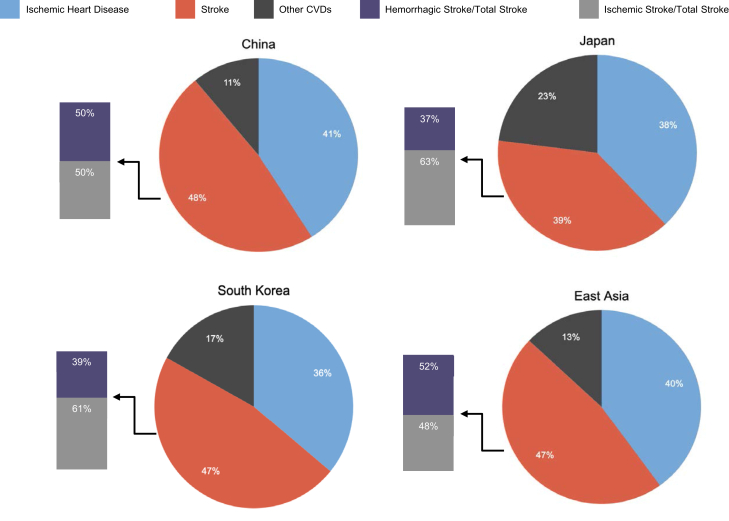
Proportion of Subtypes of CVD in Total CVD Death Percentage of total cardiovascular disease (CVD) deaths attributable to ischemic heart disease, stroke, and other CVDs in China, Japan, and South Korea, and all of East Asia. The proportion of deaths caused by stroke are further stratified into the percentage of total stroke deaths caused by ischemic or hemorrhagic stroke. Data were obtained from the open database of the Global Burden of Disease Study in the Global Health Data Exchange.

It is well established that East Asian countries have a specific epidemiological pattern of CVD. In 2019, nearly 5.2 million East Asian natives died of CVD. Overall crude CVD mortality in East Asia was 349 of 100,000 with notable differences in mortality rates across East Asian countries (Figure 1).^2^ Of the 5 major countries of this region, South Korea had the lowest crude CVD mortality rate (145 of 100,000) while North Korea had the highest (391 of 100,000). In 2019, ischemic heart disease and ischemic/hemorrhagic stroke accounted for approximately 87% of all CVD deaths in East Asia with stroke making up more than one-half of them (Figure 2).^2^ Of total deaths in East Asian countries, Japan had the lowest proportion of stroke deaths (39%), while China (48%) and South Korea (47%) had comparable rates. Although hemorrhagic strokes appeared to be slightly more frequent in East Asia overall, the proportion of hemorrhagic strokes varied significantly across the region. On average, 52% of stroke deaths were attributed to hemorrhage stroke, ranging from as low as 36% in Japan to as high as 50% in China.^2^ Although peripheral vascular disease continues to be an important contributor to CVD, the availability of peripheral vascular disease mortality data in East Asian countries remains limited.

National-level epidemiological data on ASCVD in East Asian Americans is limited, forcing us to rely on aggregated data and single center studies to estimate ASCVD prevalence and mortality rates among East Asian Americans. Based on the 2018 NHIS (National Health Interview Survey) conducted by the Centers for Disease Control and Prevention, the age-adjusted prevalence of stroke among persons 18 years of age or older was similar between non-Hispanic Asian (2.7%) and non-Hispanic White (2.7%) persons, whereas the prevalence of coronary heart disease (CHD) was lower in Non-Hispanic Asian persons at 4.4% compared with 5.7% in Non-Hispanic White persons.^12^ Based on the 2019 National Vital Statistics Report conducted by the Centers for Disease Control and Prevention, the age-adjusted death rate for cerebrovascular disease was lower in non-Hispanic Asian vs non-Hispanic White persons (cerebrovascular disease: 29.3 of 100,000 vs 35.7 of 100,000).^13^ Greater differences were observed in heart disease mortality rates for non-Hispanic Asian persons compared with non-Hispanic White persons. The age-adjusted heart disease death rate was more than 2-fold lower in non-Hispanic Asian persons (mean 79.2 per 100,000 with 101.6 per 100,000 in men and 61.6 per 100,000 in women) vs non-Hispanic White persons (mean 166.4 per 100,000; 210.7 per 100,000 in men, and 129.6 per 100,000 in women).

The data highlighted in the previous text includes data for all Asian persons living in the United States (including those of South Asian origin that often exhibit very different risk profiles from East Asians), and the exact prevalence of ASCVD and its associated mortality in East Asian Americans is uncertain. Most surveys lack or have limited data disaggregating East Asian subgroups from all Asian and Pacific Islander persons. Although the U.S. Census Bureau has made great strides to ensure accurate representation of Asian immigrants in surveys through outreach programs, native-language interviews, subgroup categorization, and oversampling, limited disaggregated data is currently available for East Asian Americans from national public surveys such as the NHIS and NHANES (National Health and Nutrition Examination Survey). Generalizability of these data are challenging because some surveys require a minimum level of English proficiency.

Several investigators have published disaggregated data in East Asian immigrant subgroups. In an early cross-sectional study of 21,722 Asians living in Northern California from 2007 to 2009 and receiving care from a private health care system, the prevalence of CHD and stroke among East Asian persons (ie, Chinese, Japanese, and Korean) was 2.07% (274 of 13,181) and 0.75% (100 of 13,181), respectively. These rates were substantially lower than the prevalence of CHD (3.9%, 2,805 of 72,701) and stroke (1.2%, 846 of 72,701) among non-Hispanic White persons.^14^ A more recent 2020 study that included 51,006 East Asian persons from Kaiser Permanente Northern California with CVD risk factors, the incidence of CHD (including coronary revascularization, MI, and cardiovascular death) was 4.46% in East Asian persons compared with 6.29% in non-Hispanic White persons over a 10-year follow-up period.^15^

Three studies have examined mortality rates of ischemic heart disease and stroke for the 6 largest Asian American subgroups, including East Asian immigrants from China, Japan, and Korea and 3 other Asian subgroups (Asian Indian, Filipino, and Vietnamese).^16^, ^17^, ^18^ Two of the earlier studies used mortality data from 34 states that reported deaths for each Asian subgroup in the 2003 to 2010 Multiple Cause of Death Mortality Database from the National Center for Health Statistics.^16^^,^^17^ A more recent study by Shah et al^18^ incorporated 2017 death rates and found that age-adjusted mortality rates from ischemic heart disease are lower for non-Hispanic Asian persons compared with Hispanic and non-Hispanic White persons; however, age-adjusted mortality rates from cerebrovascular disease are comparable between Asian and non-Asian persons. Analysis of Asian subgroups reveals significant differences in mortality rates with Korean persons having the highest mortality rates among the East Asian population but much lower than South Asian persons. More contemporary data on the incidence, prevalence, and mortality associated with ASCVD for Chinese, Japanese, and Korean Americans as well as other East Asian subgroups are critical to refinement of risk assessment tools and treatment strategies.

### Prevalence of ASCVD risk factors in East Asian populations living in Asia and in the United States

Cardiovascular risk factors, including hypertension, diabetes, dyslipidemia, tobacco use, overweight/obesity, and lifestyle factors such as unhealthy nutritional practices and physical inactivity, contribute to the development of ASCVD. Understanding the prevalence of major CVD risk factors among East Asian immigrants is critical for predicting the risk of developing ASCVD. Accurate and current information on the prevalence of cardiovascular risk factors in East Asian immigrants is not only the basis for evaluation of which risk prediction models are most accurate for East Asians, but also form the basis for recalibration of these models. The following sections summarize existing data on the prevalence of cardiovascular risk factors for East Asian people living in their native countries and in the United States, respectively.

The epidemic of unhealthy lifestyles continues to drive the prevalence of ASCVD risk factors in East Asia, as evidenced by the data collected by the NCD Risk Factor Collaboration (NCD-RisC),^16^ a network of health scientists that provides rigorous and timely global data on major risk factors for noncommunicable diseases. According to the most recent data available, South Korea had the lowest age-standardized prevalence of hypertension (13.8% in men and 8% in women) and obesity (4.5% in men and 5.0% in women). Japanese people had the highest mean levels of total cholesterol (TC) (4.98 mmol/L in both men and women) and high-density lipoprotein-cholesterol (HDL-C) (1.46 mmol/L in men and 1.77 mmol/L in women), while Chinese people had the lowest mean levels of TC (4.56 mmol/L in men and 4.66 mmol/L women) and HDL-C (1.20 mmol/L in men and 1.35 mmol/L in women). The U.S. population had much higher prevalence of obesity (36.0% in men and 38.1% in women) compared with the risk factor profiles of East Asian countries.^14^^,^^19^ Unfortunately, the numbers reported in the NCD-RisC reflect percentages in the United States as an entire population, which limit our ability to compare East Asian persons and their U.S. counterparts and further highlight the need for a global effort to disaggregate data by race and ethnicity. Moreover, the aforementioned percentages of obesity defined in the United States reflect body mass index (BMI) cutoffs for obesity defined in non-Asian persons. Asian American people are more likely to have central obesity, which remains a major risk factor for the development of type 2 diabetes, metabolic syndrome, and CVD at lower BMIs than non-Asian people. Evidence supporting racial differences in fat distribution between Asian and non-Asian people has prompted the lowering of the cutoff for obesity for Asian persons to ≥27.5 and ≥25.0 kg/m^2^ rather than the cutoff 30 kg/m^2^ for non-Asian persons by the World Health Organization and other regulatory bodies (eg, Japanese Society for the Study of Obesity, Korean Society for the Study of Obesity),^20^ respectively. Thus, the estimates of obesity by the NCD-RisC may underestimate the true incidence of obesity in East Asian Americans and non-East Asian Americans.

The most recent estimates of cardiovascular risk factor prevalence in Asian Americans were published by Commodore-Mensah et al^20^ and Koirala et al,^8^ who independently evaluated the heterogeneity of CVD risk factors among U.S. Asian immigrants using a cross-sectional analysis collected from NHIS between 2010 to 2018. They compared the prevalence of CV risk factors between 474,968 White adults and 33,973 Asian immigrants including an estimated 9,753 individuals from the Asia region (including Asia Minor, China, Democratic People’s Republic of Korea [North Korea], Japan, Mongolia, and Republic of Korea [South Korea]). The remaining Asian immigrants included in the analysis were from South Asia (Afghanistan, Bangladesh, Bhutan, India, Nepal, Pakistan, Sri Lanka) and Southeast Asia (Burma, Cambodia, Indonesia, Laos, Malaysia, Philippines, Singapore, Thailand, and Vietnam) regions. Notably, the geoscheme for Asia used by the authors differs from the geoscheme defined by the United Nations and adopted by the writers of this review.

Compared with non-Hispanic White persons, East Asian immigrants were less likely to have hypertension (20.8% vs 34.3%), diabetes (6.3% vs 9.6%), dyslipidemia (21.9% vs 31.7%), or overweight/obesity (47.1% vs 64.1%). Rates of smoking were not significantly different between non-Hispanic white and East Asian Americans. Compared with South Asian immigrants, East Asian immigrants were more likely to have hypertension (20.8% vs 16.6%), but less likely to report having diabetes (6.3% vs 8.6%) or overweight/obese (47.1% vs 71.54%). The rate of dyslipidemia between East Asian immigrants and South Asian immigrants was comparable (21.9% vs 20.8%). Compared with Southeast Asian immigrants, East Asian immigrants were less likely to have hypertension (20.8% vs 31.2%), diabetes (6.30% vs 11.3%), dyslipidemia (21.9% vs 33.41%), or overweight/obesity (47.1% vs 59.8%). Physical inactivity was equally present in all Asian subgroups with over 50% of patients not meeting physical activity guidelines. Compared with non-Hispanic White persons, East Asian persons were more likely to be physically inactive, defined by the proportion of the population not meeting ACC/AHA guidelines for exercise and physical activity (50.8% vs 48.3%). Even after adjustment for age, sex, and socioeconomic factors, East Asian persons were more likely to report physical inactivity (prevalence ratio [PR] 1.14, 95% CI: 1.09-1.19) but less likely to report hypertension (PR 0.72, 95% CI: 0.67-0.77), overweight/obesity (PR 0.83, 95% CI: 0.80-0.87), diabetes mellitus (PR 0.80, 95% CI: 0.70-0.91), high cholesterol (PR 0.83; 95% CI: 0.7700.90), and current smoking (PR 0.53, 95% CI: 0.46-0.60) than their non-Hispanic White counterparts. Unfortunately, this analysis was unable disaggregate subjects by their country of origin—a critical variable to estimate the incidence and prevalence of CVD risk factors among East Asian American subgroups and essential for creating a foundation for accurate risk estimation.

### The impact of acculturation and environmental effects of ASCVD risk profiles

It is fundamental to understand the potential differences in CVD risk factors and ASCVD mortality profiles of East Asian natives and East Asian Americans before we can develop an accurate risk assessment strategy. To better account for acculturation and environmental effects, risk factor tools for East Asian immigrants may need to consider the impact of immigration history and generational status on risk factor profiles. Few studies have directly compared the profiles of CVD risk factors between Asian Americans and Asian immigrants. In the REACH (REduction of Atherothrombosis for Continued Health) registry that enrolled close to 3,000 Chinese patients with atherothrombotic disease living outside and inside mainland China, rates of obesity, hypertension, hypercholesterolemia, and diabetes were lower in mainland Chinese people than those in China Hong Kong, China Taiwan, Singapore, Western Europe, and North America.^21^ In a recent cross-sectional analysis of the Japanese population, Hirooka et al^22^ showed that Japanese Americans reported less sedentary behavior but were also less likely to engage in physical activity and formal exercise. Although the findings are based on studies conducted a decade ago, they highlight the need to account for environmental and cultural factors to create an accurate risk stratification tool for East Asian Americans. A recent study found that acculturation was associated with a heterogeneous pattern of CVD risk factors among Asian American subgroups, highlighting the need for more research to better understand these differences and to guide culturally concordant interventions.^23^

## Current State of ASCVD Risk Calculators for East Asian Populations

In the 2018 ACC/AHA Guideline on the Management of Blood Cholesterol,^24^ the PCE was used for risk assessment for primary prevention to guide the eligibility for statin therapy. It was developed by combining 4 U.S. community cohort studies, including the ARIC (Atherosclerosis Risk in Communities), CHS (Cardiovascular Health Study), CARDIA (Coronary Artery Risk Development in Young Adults), and Framingham Study. It predicts the 10-year risk of ASCVD (including nonfatal and fatal CHD and stroke). The risk factors used in this model include sex, race (non-Hispanic White, African American, or other), age, systolic blood pressure, treatment for hypertension, TC, HDL-C, smoking, and diabetes. Notably, the cohorts used in the PCE included very few Asian subjects. Moreover, these cohorts were initiated in the 1980s or earlier when CVD risks were higher or have tended to overestimate ASCVD risks caused by this secular effect. Also, as detailed in the following text, estimated 10-year ASCVD risk is generally lower in Asian American persons compared with non-Hispanic White persons. Accordingly, Asian-specific risk stratification calculators linked with clinical guidelines are needed to bridge these gaps (Central Illustration).^25^

**Central Illustration:**
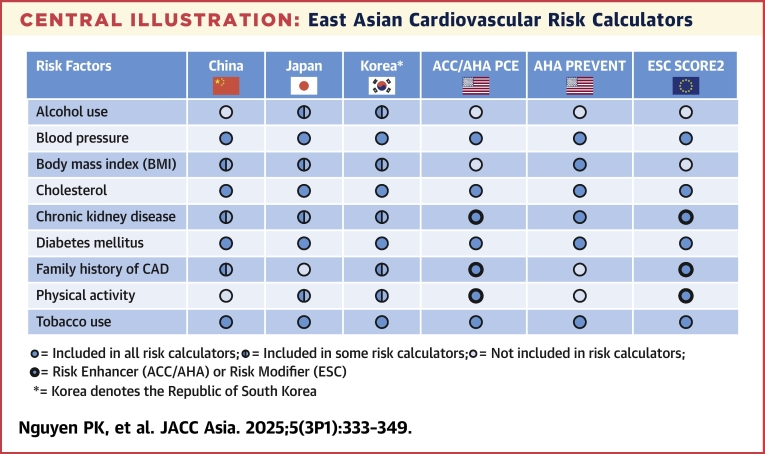
East Asian Cardiovascular Risk Calculators Comparing cardiovascular risk calculators: China, Japan, Korea, United States, and Europe. Filled circles: included in all risk calculators; half-filled circles: included in some risk calculators; open circles: not included in risk calculators; circles with bold outlines: risk enhancer (American College of Cardiology [ACC]/American Heart Association [AHA]) or risk modifier (European Society of Cardiology [ESC]). ∗Korea denotes the Republic of South Korea. CAD = coronary artery disease.

### ASCVD risk prediction in China

Several risk scores have been evaluated for ASCVD risk prediction in China. In 2003, the CMCS (Chinese Multi-provincial Cohort Study) of 30,121 adults (ages 35 to 64 years) evaluated the performance of the Framingham CHD risk score with equations derived from CMCS.^25^ The investigators found that when compared directly and after recalibration, the original Framingham equation significantly overestimated absolute CHD risk in the CMCS cohort, which was mainly driven by differences in the mean CHD risk and the levels of major risk factors between the 2 cohorts. Specifically, the 10-year CHD event rates were 8.0% and 2.8% in Framingham men and women, respectively, compared with 1.5% and 0.6% in the CMCS men and women. With respect to cardiac risk factors, men and women in the Framingham cohort had higher TC and lower HDL-C, respectively, than those in the CMCS cohort. However, men in the Framingham cohort had lower and women had higher smoking rates in comparison with men and women in the CMCS cohort. Importantly, a recalibration of the Framingham Risk Score (FRS) equation (ie, the mean risk of CHD and mean levels of risk factors derived from Framingham cohort), performed by replacing the corresponding estimations from CMCS cohort, substantially improved the accuracy of risk prediction.^24^ Even after recalibrating Framingham risk calculators, the China-MUCA (Multicenter Collaborative Study of Cardiovascular Epidemiology) cohort study reported that CHD risk for both men and women was still substantially overestimated.^26^ More recently, another large cohort study, China-PAR (Prediction for ASCVD Risk in China) project, found that the PCE had low discrimination ability and poor calibration for Chinese men.^27^ These findings highlighted the importance of developing CVD risk prediction models based on data from China cohort studies.

Based on data from the CMCS cohort study, the first sex-specific ASCVD risk prediction equations and stratification algorithms were published in 2003 and subsequently updated in 2018.^28^^,^^29^ The China-MUCA study and China-Par project developed and published risk predictive models to estimate 10-year risk ASCVD in Chinese people.^26^^,^^27^ A comparison of these risk prediction models is shown in Table 1. After the development of 10-year ASCVD risk equations from large, long-term cohort studies, CMCS and China-PAR cohort studies were utilized to create lifetime ASCVD risk prediction models for young and middle-aged people.^29^^,^^30^ The lifetime ASCVD risk prediction tool can identify those with lower 10-year ASCVD risk but higher lifetime risk, thereby, facilitating earlier prevention intervention, including motivating lifestyle modifications for these individuals. All of these studies developed either categorized algorithms of risk classification by flowcharts, risk scoring systems, or web-based risk calculators to facilitate the application of risk assessment into clinical practice and for patient education.^26^, ^27^, ^28^

**Table 1:** ASCVD Risk Assessment Tools in China

First Author	Study Population	Predictors (Other Than Age and Sex)	Outcomes	Duration of Risk Prediction	Validation	Risk Tools
Liu et al^25^	30,121 men and women aged 35-64 y	SBP, TC, HDL-C, smoking status, diabetes	CHD	10 y	Internal	Equations
Wu et al^26^	11,336 men and women aged 35-59 y	SBP, TC, smoking status, diabetes, BMI	ASCVD	10 y	Internal External	Risk scores
Yang et al^27^ and Wang et al^28^	27,020 men and women aged 35-74 y	SBP, TC, HDL-C, smoking, diabetes, WC, geographic region, urban/rural, family history of ASCVD	ASCVD Stroke	10 y Lifetime	Internal External	Equations and web-based risk calculator
Wang et al,^28^ Wang et al,^29^ and Wang et al^82^	30,121 men and women aged 35-64 y	SBP, LDL-C, HDL-C, smoking status, diabetes	ASCVD	10 y Lifetime	Internal	Equations, risk charts, and flowcharts of risk classification

ASCVD = atherosclerotic cardiovascular disease; BMI = body mass index; CHD = coronary heart disease; HDL-C = high-density lipoprotein cholesterol; LDL-C = low-density lipoprotein cholesterol; SBP = systolic blood pressure; TC = total cholesterol; WC = waist circumference.

Since 1999, ASCVD risk stratification-based clinical decision making has been recommended in China by relevant CVD prevention practice guidelines to inform treatment strategies and targets for risk factor control.^31^, ^32^, ^33^ The 1999 Chinese guidelines for the management of hypertension was the first to employ risk assessment to guide decision-making for blood pressure treatment.^31^ The risk classification criteria recommended by the World Health Organization/International Society of Hypertension blood pressure guideline were used because no risk assessment tools were available in China at that time. The 2007 Chinese guidelines on prevention and treatment of dyslipidemia in adults was the first to use ASCVD risk classification based on risk prediction models developed from Chinese cohort studies (CMCS and China-MUCA) and was notable for its heavy weighting of hypertension.^32^ Risk classification charts were updated based on new risk prediction equations and lifetime risk estimation developed from CMCS data for the 2016 Chinese management of dyslipidemia guideline for adults and 2017 China guideline for CVD prevention.^34^^,^^35^

In 2020, the Chinese Guideline on the Primary Prevention of Cardiovascular Diseases further modified their recommendations for risk classification by adding chronic kidney disease stage 3/4 to diabetes, age >40 years, and very high low-density lipoprotein-cholesterol (LDL-C) (≥4.9 mmol/L) as high-risk conditions.^33^ Ten risk-enhancing factors were added, including coronary artery calcium (CAC) score ≥100 Agatston units. CAC is well-established as a risk stratifying tool among Chinese adults from a large study from China of 4,425 patients with suspected coronary disease scanned for presence of CAC and coronary plaque.^36^ At 3 years of follow-up, the risk of major adverse cardiac events increased with higher CAC scores (CAC 0, 2.1%; CAC 1 to 100, 12.9%; CAC 101 to 400, 16.3%; and CAC >400, 33.8%; log-rank *P <* 0.001). The C-statistics improved with addition of CAC and plaque characteristics over traditional risk factors alone: 0.71 for clinical risk factors, which improved to 0.82 by adding CAC and further improved to 0.93 by adding coronary computed tomography angiographic (CTA) plaque information (both *P <* 0.001). In addition, the U.S. Multiethnic Study of Atherosclerosis showed CAC strongly predicted future ASCVD events for Chinese American participants (as well as other racial/ethnic groups) over 10 years, with those having CAC scores >100 identified to have an ASCVD risk ≥7.5% making them eligible for initiation of statin therapy.^34^^,^^35^^,^^37^ Recently, a new risk classification system for secondary prevention was issued by the Chinese Society of Cardiology in an expert consensus statement. Instead of considering all patients with ASCVD as a very high-risk group in previous guidelines, this statement further identified a subset at extreme ASCVD risk for more intensive lipid lowering treatment.^37^

Although country-specific risk assessment tools are readily available in China, significant challenges remain. Most of the cohort studies collected baseline information in the 1990s, but there have been changes in risk factor prevalence among more contemporary target populations. Risk prediction models need to be recalibrated, and some studies have started to evaluate these older equations with data collected from large contemporary cohorts. For instance, Liu et al^38^ recently applied the PCE and China-PAR algorithms to an electronic health record cohort of 226,406 participants and showed that although both models had good discrimination, the China-PAR model substantially underpredicted risk, and the PCE overpredicted risk in men and underpredicted risk in women, although recalibration improved this. Additionally, although most of the risk enhancing factors increased ASCVD risk in observational studies, few studies in China showed that these risk-enhancing factors, except for CAC score, could significantly improve performance of risk calculators. Several new risk factors have been considered as potential contributors to residual risk in Chinese people, including small density LDL, cholesterol-overloaded HDL particles,^39^ and higher circulating levels of PCSK9.^38^^,^^40^, ^41^, ^42^ However, the practical use of these new risk factors in individual based ASCVD risk prediction in Chinese is uncertain. Although guidelines generally recommend risk assessment to drive prevention and treatment strategies, adherence to these recommendations are low in clinical practice,^43^ especially in primary care settings.^44^ Furthermore, cost-effectiveness and feasibility of more complex and accurate risk assessment tools for clinical application are unknown. Moreover, no studies to date have evaluated if ASCVD risk prediction calculators developed from Chinese cohorts can be applied to people living in other East Asian countries or Chinese persons living abroad, including Europe and the United States.

### ASCVD risk prediction in Japan

Since 1960, when the first epidemiologic studies of CVD were performed in Japan, high stroke mortality rates but low CHD mortality rates were observed.^42^^,^^45^ Although stroke mortality has significantly improved in subsequent decades, CHD mortality remains low compared with Western populations. The 2012 Japan Atherosclerosis Society (JAS) Guidelines for Prevention of Atherosclerotic Cardiovascular Diseases was the first guideline to introduce absolute risk assessment of ASCVD and used the NIPPON DATA80 risk chart to predict 10-year CHD mortality.^40^ Similarly, the 2019 Japanese Society of Hypertension incorporated absolute risk assessment as a reference to inform blood pressure treatment thresholds and management.^41^

In the 2017 JAS guideline,^42^ the Suita score was able to accurately estimate the absolute incidence of CHD by incorporating demographics and risk factors including age, sex, smoking, blood pressure level, HDL-C, LDL-C, impaired glucose tolerance, and family history of premature CHD.^45^ The Suita score was chosen from 10 different published risk prediction scores in Japan where internal validation was carefully performed. Although attempts were made to validate the predictive model for the guidelines in an external population, even within Japan, cardiovascular disease risk differs between urban and nonurban areas. Thus, it was extremely difficult to set a standard population for external validation.^42^

Based on the algorithm for ASCVD risk assessment, individuals are first screened to determine if they should be categorized as primary or secondary prevention candidates. They are also evaluated for high-risk comorbidities including diabetes, chronic kidney disease, noncardiogenic stroke, and peripheral artery disease. If none of these conditions are present, the Suita score is calculated and individuals are stratified into low-, moderate-, and high-risk categories. The LDL-C targets in primary prevention are set at <160 mg/dL for low risk, <140 mg/dL for moderate risk, and <120 mg/dL for high risk, respectively. The LDL-C target for patients with established CHD is <100 mg/dL, but if they also have a history of familial hypercholesterolemia or acute coronary syndrome, then a lower target LDL-C level of <70 mg/dL should be considered. Patients with both diabetes and ASCVD should also be treated to an aggressive LDL-C goal of <70 mg/dL.

It is important to note that the absolute ASCVD risk estimated by the Suita score only includes CHD and not stroke, unlike the PCE and SCORE2 risk calculators that include both. In Japan, however, cerebral hemorrhage accounts for a high proportion of strokes, whereas the percentages of lacunar, cardioembolic, and atherothrombotic strokes are almost equivalent,^46^^,^^47^ with the first 2 not being associated with hypercholesterolemia.^46^ Although Japanese people have a high incidence of stroke, they have a relatively low proportion of stroke phenotypes associated with dyslipidemia, and therefore it has been difficult to use “stroke” when setting lipid management targets. In contrast, hypercholesterolemia is a strong risk factor for atherothrombotic infarction. Accordingly, the new 2022 JAS guideline, uses a recently published risk score from the Hisayama study that predicts incidence of the combined outcome of CHD and atherothrombotic cerebral infarction for individual risk assessment.^48^ A summary of ASCVD calculators used in Japan is shown in Table 2.

**Table 2:** ASCVD Risk Assessment Tools in Japan

First Author	Study Population	Predictors (Other Than Age and Sex)	Outcomes	Duration of Risk Prediction, y	Validation	Risk Tools
Iso et al^52^	10,334 men and 19,542 women aged 40-69 y	hsCRP SBP, DBP, DM, HDL, LDL, TC, alcohol, BMI, smoking habits, and medications	ASCVD	10	Internal	Odds ratio
Nippon Data80 et al^40^	4,098 men and 5,255 women age 30 y or higher	SBP, smoking, TC and glucose levels	ASCVD	19	Internal	Risk charts, Cox proportional hazard models
Hirai et al^45^	14,072 men and 21, 307 women Women aged 40-79 y	SBP, DM, HDL, LDL, CKD stage, smoking habits	CHD	3	Internal	Suita risk score
Honda et al^83^	1,026 men and 1,428 women aged 40-84 y	SBP, DM, HDL, LDL, proteinuria, smoking habits, and regular exercise.	ASCVD	24	Internal	Cox proportional hazard model

CKD = chronic kidney disease; hsCRP = high sensitivity C-reactive protein; other abbreviations as in Table 1.

The new JAS guideline also reviews the application of imaging and physiological measurements for subclinical atherosclerosis detection and their additive value to risk prediction models. These markers include the following: 1) periventricular hyperintensity or deep and subcortical white matter hyperintensity by brain magnetic resonance imaging; 2) carotid intima-media thickness and plaque characteristics; 3) CAC; 4) pulse wave velocity; 5) cardio-ankle vascular index; and 6) ankle-brachial index (ABI). The authors reviewed whether these potential risk enhancers improve the predictive ability of traditional risk factors for the development of ASCVD in a primary care setting. A meta-analysis of Japanese studies showed that brachial-ankle pulse wave velocity (baPWV) improves risk prediction,^49^ but the model was not generated from a Japanese population. Therefore, the applicability to set baPWV cutoff values for Japanese people remains elusive. Other markers, including magnetic resonance imaging, carotid intima-media thickness, and plaque characteristics, and CAC score still lack evidence for risk prediction, and ABI did not enhance risk prediction models.

For high-risk individuals, such as stable patients with suspected CHD, the addition of CAC score to a risk prediction model that employs traditional risk factors significantly improved CHD risk classification.^50^ Given the high availability of computed tomography (CT) scanners in Japan (highest per capital in the world, 115.7 per million people in 2020),^51^ many studies have been conducted to probe the additive value of coronary CT angiography and CAC score for symptomatic patients. However, use of these imaging modalities for comprehensive risk assessment have not been performed. This is also true for other imaging and physiological measurements, such as magnetic resonance imaging, carotid intima-media thickness, plaque characteristics, baPWV, and cardio-ankle vascular index. Biomarkers such as C-reactive protein,^52^ small dense LDL,^53^ and modified lipoproteins (LOX-1 ligands containing apolipoprotein B,^54^ malondialdehyde-modified LDL^55^) have also been validated in epidemiological and clinical studies in Japan and have been found to refine ASCVD risk when added to classical risk factors. However, they have only been assessed at the individual level, and without validation in population-based cohorts, are difficult to incorporate into risk prediction models. They have limited clinical application since they are not routinely measured at clinic visits.

Since the establishment of universal health coverage for all Japanese people in 1961, there are limited incentives to use imaging modalities such as CT scans to enhance systematic risk prediction or to evaluate the cost-effectiveness of these tests, especially for high-risk individuals. Future directions should focus on determining whether biomarkers or subclinical atherosclerosis detection can improve risk assessment for low- and intermediate-risk people.

### ASCVD risk prediction in the Republic of Korea (South Korea)

Early Korean studies attempted to recalibrate risk assessment tools developed in Western populations because they overestimated the absolute risk of ASCVD in the Korean population.^56^, ^57^, ^58^, ^59^ Later studies used Korean data to develop risk prediction models for stroke, coronary artery disease, or ASCVD.^59^, ^60^, ^61^, ^62^, ^63^, ^64^, ^65^, ^66^ The National Health Insurance Service, which operates a free health screening program for all Korean adults, provides risk assessment for CVD and diabetes based on individualized data. Private health screening centers and research institutes are also using disease prediction on common chronic diseases, including ASCVD, diabetes, cancer, and Alzheimer’s disease. However, ASCVD risk prediction assessment has not yet been adopted as the primary tool for risk assessment in clinical practice guidelines.^67^ A summary of available ASCVD risk assessment tools in Korea is provided in Table 3.

**Table 3:** ASCVD Risk Assessment Tools in Korea

First Author	Study Population	Predictors (Other Than Sex and Age)	Outcomes (Follow-Up)	Duration of Risk Prediction, y	Validation	Risk Tools Provided
Jee et al^60^	777,502 men and 446,238 women aged 30-84 y	SBP, TC, diabetes, smoking, activity, BMI, alcohol	Stroke	13	Internal	Equation, risk score table
Jee et al^66^	164,500 men and 104,310 women aged 30-74 y	BP, TC, diabetes, smoking, (HDLC, LDLC, TG; optional)	CHD	12	Internal	Equation, HR
Park et al^84^	3,135 men and 2,425 women aged 43-63 y	Hypertension, diabetes, SBP, DBP, TC, HDLC, LDLC, smoking, atrial fibrillation, white blood cells, creatinine, hemoglobin A1c	ASCVD	3	Internal	HR
Jung et al^59^	119,715 men and 80,295 women aged 40-79 y	SBP, TC, HDLC, DM, smoking, antihypertensive	ASCVD	10	Internal	Equation
Cho et al^61^	152,076 men and 144,354 women aged 40-79 y	BMI, SBP, DBP, TC, FBG, smoking, activity	ASCVD	10	Internal and External	Machine learning algorithm
Cho et al^63^	222,998 individuals aged 40-70 y	SBP, DBP, TC, HDLC, LDLC, TG, FBG, GGT, GFR, proteinuria, BMI, WC, smoking, alcohol, activity, antidiabetic, antihypertensive, statins, family history of CHD, family history of stroke	ACS + stroke	8	Internal	HR, nomogram
Choi et al^64^	10,412,947 men and 11, 168, 849 women Aged 40-79 y	SBP, TC, HDLC, diabetes, smoking, antihypertensive, (+8 optional markers)	ASCVD	5	Internal	Machine learning algorithm
(Unpublished)	43,798 individuals aged 40-79 y	SBP, TC, HDLC, diabetes, smoking, antihypertensive	ASCVD	10	Internal and external	Equation, risk chart

ACS = acute coronary syndrome; DBP = diastolic blood pressure; FBG = fasting blood glucose; GFR = glomerular filtration rate; GGT = gamma-glutamyl transferase; TG = triglycerides; other abbreviations as in Table 1.

The Korean Society of Lipid and Atherosclerosis recently released the fifth edition of the Korean Guidelines for the Management of Dyslipidemia. Compared with the fourth edition, the revised guidelines lowered the LDL-C target level for patients with CHD from <70 to <55 mg/dL.^68^^,^^69^ The risk classification of people with diabetes was also subdivided based on recent Korean data,^70^ with the LDL-C target value lowered in selected high-risk diabetes groups.^71^ Lower LDL-C targets are also recommended for patients with significant carotid artery stenosis or abdominal aortic aneurysm. Although risk classification based on the presence of specific risk factors remained, it is noteworthy that the fifth KSOLA guidelines introduced, for the first time, an optional recommendation to use a specific ASCVD prediction model. The guidelines noted that "ASCVD risk scores based on the Korean Genome and Epidemiology Study (KOGES) cohort can be used as a risk enhancer in low- or moderate-risk groups. ^69^ The reason why it is recommended to be used only in low- to moderate-risk groups is that the absolute incidence of ASCVD is very low in Koreans, so if they rely only on the ASCVD prediction model, some patients high-risk groups with major modifiable risk factors may not be identified for treatment.^69^ The Korean Society of Hypertension (KSH) released the 2022 KSH Guidelines for the Management of Hypertension without significant changes in risk classification compared with the previous 2018 KSH guideline. Assessment of CVD risk and blood pressure targets are still based on the presence or absence of clinical ASCVD or the number of established cardiovascular risk factors. The KSH guidelines acknowledge the importance of accurate individual risk assessment for selecting the best ASCVD prevention strategy and recommend the use of risk assessment tools but does not endorse a specific risk prediction model.^69^^,^^72^

There are several reasons why ASCVD risk prediction is mainly of academic interest and is not widely accepted in clinical practice guidelines in Korea. Because most of the ASCVD risk prediction models developed in Korea are based on data from limited health screening centers, there is criticism about the lack of adequate representation. Additionally, the ASCVD risk prediction models were mostly validated internally without external validation in a diverse cohort population. Furthermore, there is insufficient evidence demonstrating the clinical efficacy and cost-effectiveness of preventive pharmacological interventions. These deficiencies make it difficult to recommend an ASCVD risk prediction model in daily clinical practice.^67^ Risk factors in the Korean ASCVD risk prediction models are similar to those used in other countries. The major risk predictors include sex, age, blood pressure, smoking, diabetes, and TC. It is notable that many of the Korean prediction models only use TC instead of LDL-C and HDL-C because Korea's general national health screening program often only measures TC levels.^59^, ^60^, ^61^^,^^66^ In addition, the incidence of CHD is low, and LDL-C and TC levels are strongly correlated to risk in the Korean population while the predictive power of TC is not inferior to LDL-C plus HDL-C levels (54,64). Efforts to use calculators, including the SCORE2 (Systemic Coronary Risk Evaluation 2) and SCORE2-OP (SCORE2-Older persons), resulted in an underestimation risk in young Korean men and women (aged 40-59 years) and overestimation in older individuals, highlighting the need for a region-specific calculator.^71^

The most recently developed Korean ASCVD prediction model is based on data from over 150,000 participants in the Korean Genome and Epidemiology Study. The risk predictors are similar to previous models, but this model includes large-scale prospective cohort data and was validated both internally and externally. In addition, the new model may have better clinical utility because the incidence of CVD in Koreans is rapidly increasing. Although ASCVD risk scores are not officially adopted in clinical practice guidelines, many CVD risk prediction studies have been published in Korea utilizing new biomarkers and imaging modalities.^73^, ^74^, ^75^, ^76^, ^77^, ^78^, ^79^ Several of them have demonstrated improvements in ASCVD predictive power using CAC or CTA and employing machine learning methods. Earlier studies cross-sectionally compared CAC and Western-derived ASCVD risk scores among asymptomatic individuals and reported that high CAC levels are also observed among some Korean persons with low to moderate ASCVD risk.^73^^,^^75^ These findings indirectly support the usefulness of CAC-based risk classification. Later studies added more direct evidence of CAC score in the risk classification of asymptomatic Korean adults. The CONFIRM (COronary CT Angiography EvaluatioN For Clinical Outcomes InteRnational Multicenter) study evaluated the clinical utility of CAC and coronary CTA in Korean as well as European/American populations.^72^ The CONFIRM study reported that CAC improved risk stratification and provided incremental value beyond FRS for predicting major adverse cardiac events.^72^ Another Korean study reported that the addition of CAC to FRS improved risk prediction for CVD mortality in young adults but not in older individuals.^80^ The predictive value of coronary CTA was also evaluated in combination with CAC. In a 2-year follow-up study of Koreans participating in health screens, both CAC and coronary artery stenosis on coronary CTA are independent predictors of CVD outcomes. However, when CAC and coronary CTA stenosis were evaluated simultaneously, CVD risk was associated only with stenosis on coronary CTA but not with CACS.^74^ In the CONFIRM study, coronary CTA provides incremental predictive value for asymptomatic individuals with moderate CAC scores (100-400), but not for lower or higher CAC scores.^76^ There are also studies that evaluated the usefulness of fundoscopy.^78^^,^^79^ A Korean study found that virtual assessment of CAC estimated from deep learning analysis of retinal photographs is comparable to CT-measured CAC in predicting CVD events and improves current risk stratification for cardiovascular events.^79^ If further validated, fundoscopy plus deep learning algorithms have the potential to serve as a cost-effective and radiation-free alternative to measuring CAC, particularly in resource-limited settings. Collectively, these studies suggest that the inclusion of imaging strategies to improve the accuracy of risk calculators such as SCORE2 and SCORE2-OP in young and old Koreans, respectively.^81^

### Future Directions and Conclusions

In this overview of ASCVD risk assessment in East Asian countries, specifically China, Japan, and South Korea, ASCVD risk is significantly overestimated, in particular CHD, when applying calculators developed in the United States including the FRS and PCE. Unlike Europe and the United States, incidence of CHD is much lower while stroke rates are higher in Japan, Korea, and China. Studies to recalibrate these risk scores have been unsatisfactory, resulting in each country developing their own risk prediction scores based on epidemiologic studies using native cohorts and traditional risk factors.^82^, ^83^, ^84^ Unfortunately, many of these national risk prediction scores lack external validation and generalizability. Furthermore, there are challenges to implementation and adherence of guidelines in clinical practice. Application of additional risk enhancers such as CAC score and biomarkers have not been extensively studied to determine their ability to improve risk prediction models. Moreover, the utility of using specific biomarkers in risk prediction algorithms may be limited if they are not routinely measured in clinical practice. Each country's risk score was developed using Western risk calculators as a framework, and no cross validation has been performed between among China, Japan, and South Korea, nor among a larger aggregate that includes other East Asian countries. This may provide an opportunity to develop a more refined regional risk score and new risk indices through collaboration given similarities in ASCVD prevalence, disease characteristics, and lifestyles.

Although the current landscape of ASCVD risk prediction tools in East Asian countries is improving, there is still significant need for refinement. External validation, implementation, and adherence are significant challenges. Consequently, there is a great need for multinational approaches for the conduct of registries and clinical trials in East Asian countries and beyond. Region-specific standardized protocols for risk factor assessment and ASCVD outcomes should be created to improve generalizability of these risk prediction models. As discussed previously, although United States-based scoring algorithms can discriminate ASCVD risk reasonably well in Asian populations, when recalibrated,^85^ they substantially overestimate actual risk in Asian cohorts. The Asia Pacific Cohort Studies Collaboration (APCSC) risk equation for predicting 8-year risk of a major cardiovascular event was the first unified attempt to develop such a model, but only involved China, Japan, Korea, and Singapore,^86^ and having been conducted 2 decades ago, is in need of further updating in a larger set of Asian-Pacific countries.

The new risk prediction models being developed in East Asian countries should standardize the definition of ASCVD and should include CHD, stroke, and PAD. Moreover, future calculators should also consider prediction of other CVD conditions, notably heart failure and atrial fibrillation given their important burden among CVD conditions. Given the higher rates of cerebrovascular disease relative to CHD in the region, standardization of these risk calculators may promote better opportunities for cross-validation. As highlighted in the previous text, risk-enhancing factors have been validated in Western populations but may perform differently in Asian cohorts. Therefore, identification of risk-enhancing factors unique to East Asian populations should be characterized to improve risk classification. Because they are easy to perform and easily understood by patients, clinical implementation of race- and ethnic-specific ASCVD risk calculators should not be difficult and welcomed by providers who yearn for more accurate risk estimators for patient subgroups. Although they may not be readily available, established risk modifiers such as subclinical atherosclerosis detected by noninvasive imaging such as CAC, carotid ultrasound, and ABI should be studied more extensively in East Asian countries and may improve risk refinement with the caveat that many patients receiving these imaging studies may be at higher risk for ASCVD, which results in selection bias and may affect the accuracy of risk assessment. At the 2023 European Society of Cardiology meeting, a new regional risk prediction model for Asia, SCORE2-ASIA, was revealed. This new risk assessment tool for Asia will be recalibrated using multiplication factors based on average CV risk in each Asian region (categorized as low-moderate, high, or very high risk). Once these risk prediction models are both internally and externally validated, they could be tested in Asian American cohorts.

The challenge to validating these risk models in U.S. cohorts is the lack of participation of East Asian Americans in clinical trials and their low representation in national health surveys. More importantly, disaggregation of Asian Americans participating in clinical trials and surveys is lacking. Although the NHIS provides disaggregated Asian-American subgroups, this data is only available for 3 of the largest Asian subgroups (Asian Indian, Chinese, and Filipino persons) because of the small number of participants for other Asian subgroups (mostly Japanese, Koreans, and Vietnamese persons). Although the NHANES oversampled for Asian American participants for several years, access to disaggregated Asian subgroup data is restricted. While East Asian countries represent more than one-quarter of the world’s population, representation of subjects in global clinical trials that inform international practice guidelines is far less. In the REDUCE-IT (Reduction of Cardiovascular Events With Icosapent Ethyl–Intervention Trial) trial of icosapent ethyl,^87^ only 3% of patients were from the Asia-Pacific region while 12% of participants were from Asia in the ODYSSEY OUTCOMES (Evaluation of Cardiovascular Outcomes After an Acute Coronary Syndrome During Treatment With Alirocumab) trial of alirociumab.^88^ One spotlight is the American College of Cardiology NCDR (National Cardiovascular Data Registry) that recently began to include disaggregated Asian and Hispanic subgroups for many of its registries.

Until there is a critical mass of Asian American participants in clinical trials and registries, it will be exceedingly difficult to create a more refined ASCVD risk assessment tool for East Asian Americans or other Asian American subgroups such as South Asian Americans. A call to action through advocacy and federal legislation that requires health care systems and national health agencies, including the Centers for Disease Control and Prevention, to collect and report disaggregated Asian American subgroup information may be necessary. Recently, the National Heart, Lung, and Blood Institute announced a new epidemiological cohort study to address key population health gaps for Asian Americans, Native Hawaiians, and Pacific Islanders.^89^ The 7-year study (MOSAAIC [Multi-ethnic Observational Study in American Asian and Pacific Islander Communities) was launched in August 2023 and will recruit 10,000 adults, ages 18 to 64 years, from 5 U.S. sites. It will focus on cardiovascular health as well as other conditions including lung health, mental health, and social determinants of health in individuals who self-identify as having ancestral background from East Asia, South Asia, or Southeast Asia; or who self-identify as Native Hawaiian and/or Pacific Islander. This study is a major step forward to enhance research efforts of Asian subgroups in the United States and may help to accelerate initiation of studies for these populations.

## Funding Support and Author Disclosures

Dr Nguyen has received support from the American Heart Association (AHA 23SCISA1145819). Dr Okamura has received funding support from Grant-in-Aid for Scientific Research (A) (21H04854) and Health and Labour Sciences Research Grants, Japan (Comprehensive research on lifestyle related diseases including cardiovascular diseases and diabetes mellitus) (24FA1002). Dr Wong has received funding through his institution from Amgen, Novartis, Novo Nordisk and Regeneron; and is a consultant for Amgen, Heart Lung, Ionis, and Novartis. Dr Yang is supported by the UW Medicine Asian Health Initiative and the Carl and Renée Behnke Endowed Chair for Asian Health; has received research funding from Microsoft Research; is a stockholder and advisory board member for Measure Labs; and is a consultant for Idorsia, Genentech, and Mineralys. All other authors have reported that they have no relationships relevant to the contents of this paper to disclose.
